# Neural Pathways in Allergic Inflammation

**DOI:** 10.1155/2010/491928

**Published:** 2011-02-09

**Authors:** L. Mirotti, J. Castro, F. A. Costa-Pinto, M. Russo

**Affiliations:** ^1^Department of Immunology, Institute of Biomedical Sciences, University of São Paulo, Brazil; ^2^Department of Pathology, School of Veterinary Medicine and Animal Science, University of São Paulo, Brazil

## Abstract

Allergy is on the rise worldwide. Asthma, food allergy, dermatitis, and systemic anaphylaxis are amongst the most common allergic diseases. The association between allergy and altered behavior patterns has long been recognized. The molecular and cellular pathways in the bidirectional interactions of nervous and immune systems are now starting to be elucidated. In this paper, we outline the consequences of allergic diseases, especially food allergy and asthma, on behavior and neural activity and on the neural modulation of allergic responses.

## 1. Introduction

**Figure 1:**
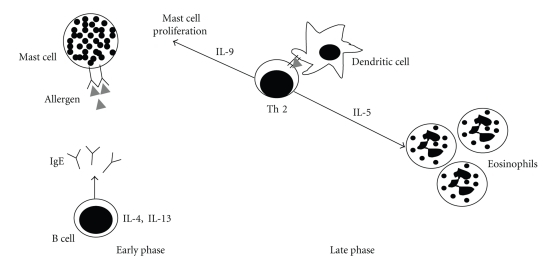
Early phase and late phase of allergic hypersensitivity. Upon allergen challenge, sensitized individuals can present two distinct phases: the early phase, which is characterized by mast cell degranulation and release of inflammatory mediators triggered by cross-linking IgE antibodies present on mast cells membranes, and the late phase, that is characterized by the infiltration of Th2 cells that interact with dendritic cells releasing type 2 cytokines responsible for tissue mast cell proliferation and eosinophil recruitment.

The prevalence of allergic diseases is continuously increasing. It is estimated that approximately one-third of the general population is affected by allergic diseases. Asthma, food allergy, dermatitis, and systemic anaphylaxis are amongst the most common allergic diseases. The myriad of symptoms observed may involve the airways, the gastrointestinal (GI) tract, the skin, and other systems [1]. The underlying mechanisms involved in the pathophysiology of classic allergic reactions have been well characterized in humans and experimental animal models [2–4]. Allergic reactions might progress in two distinct phases: an early response, which is characterized by mast cell degranulation and release of inflammatory mediators as a consequence of IgE antibodies cross-linked to their high-affinity receptors (Fc*ε*RI) expressed on mast cells membranes, and a late phase response, characterized by a T-helper type 2 (Th2) response, with an increased secretion of cytokines such as IL-4 and IL-13, which stimulate B cells to synthesize IgE; IL-5, necessary for eosinophilic inflammation; IL-9, which stimulates mast cell proliferation [5] (see Figure 1).

There is robust evidence indicating interactions between the immune and nervous systems [6, 7]. There are three types of interactions between the immune system and the central nervous system (CNS); first, the immune system regulates the CNS; second, the CNS drives immunity; third, the CNS acts reciprocally with the immune system. It is well established that these systems, along with the endocrine system, share receptors for cytokines, neurotransmitters, hormones, and neuropeptides. Molecules previously reported as products of a particular system were shown to be more broadly synthesized, such as cytokines being synthesized in the CNS and hormones such as ACTH and TSH being produced by lymphoid cells [8–14].

This paper will focus on the consequences of allergic diseases, especially food allergy and asthma, on behavior and neural activity and on the bi-directional interaction between immune and nervous systems that culminates with neural modulation of allergic responses.

## 2. Neural Activity in Allergy

A plethora of epidemiological and clinical data suggests higher incidence of anxiety and increased emotional reactivity in individuals suffering from allergies [15–21]. In studies of food allergy, specifically, it has been shown that the prevalence of anxiety or depression is higher in adults with food allergy than in nonhealthy controls with lactose intolerance or in healthy controls [22]. Increased anxiety levels were also associated with food allergy in adolescents [23], and other authors have reported that food allergic children expressed higher levels of anxiety and fear associated with managing their allergy than children with diabetes [24]. Nevertheless, other studies have failed to find association between food allergy and behavioral changes [25, 26]. Asthmatics in crisis also experience changes in emotional status and increased levels of anxiety [27]. Conversely, sadness, stress, and other nervous stimuli can precipitate acute symptoms of asthma [28]. A study using functional magnetic resonance imaging has shown that the activity in the anterior cingulate cortex and insula, in response to asthma-relevant emotional stimuli, is associated with inflammation markers and airway obstruction in asthmatic subjects exposed to antigen [29]. 

Neuroimmune studies with humans are difficult to be conducted due to the challenge on achieving a precise diagnostic of allergy, the large range of allergic symptoms and severity, artifact of referral bias in population studies, the implication of putatively unrelated psychological factor, psychosomatic aspects of the disease, and ethical issues involved in submitting allergic patients to experimental contact with the allergen [20, 25, 26, 30]. 

In animal models of allergy, some of the setbacks of behavioral studies can be circumvented, and important findings have been achieved in this area in the last few years. The pioneer work evidencing behavioral changes as consequence of allergic reactions was published by Cara et al. [31]. It was shown that ovalbumin- (OVA-) allergic mice avoid drinking the otherwise preferred saccharin-sweetened solution containing the allergen (OVA) [31]. The protocol used to test the feeding behaviour was based on a two-bottle preference test, in which control or OVA-sensitized animals received water and sweetened OVA-solution in two separate bottles during 24 hours, with no previous conditioning or learning sessions. The immunological aversive phenomenon, known as food aversion, was shown to be abolished by the induction of immunological tolerance [31]. It was also demonstrated that food aversion can be transferred from OVA-allergic to naïve mice by passive transfer (injection of hyperimmune serum) or by adoptive transfer of spleen cells [32]. The immunological aversive behavior was shown to be specific, since peanut- or wheat-sensitized mice, when offered with a mixture of the grains *in natura*, avoided the ingestion of grains containing the allergen that they were sensitized but not other grains [33].

**Figure 2:**
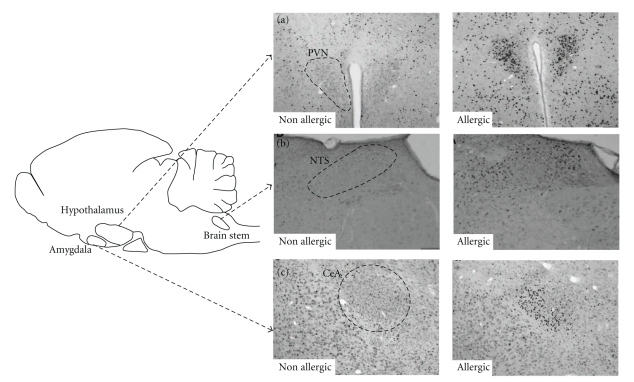
Activation of specific brain areas by c-*fos* expression. Representative brain coronal sections of nonallergic (nonsensitized) and allergic (OVA-sensitized) mice after (oral or nasal) challenge with OVA. Fos staining in neurons of (a) the paraventricular nucleus of the hypothalamus (PVN), (b) nucleus of the tract solitary (NTS), and (c) central nucleus of the amygdala (CeA). Adapted from Basso et al. 2004 [36] and Costa-Pinto et al. [47].

In view of what is known about food and behavior, including the evolutionary aspects involved with taste recognition [34], a finely constructed system of communication between the digestive system and the brain is entirely plausible. In line with this assumption, it was further demonstrated that OVA-sensitized mice orally challenged with the allergen present increased levels of anxiety, evidenced by shorter time of exploration in the open arms of an elevated plus maze and strong activation of specific brain areas, evidenced by enhanced c-*fos* expression in the paraventricular nucleus of the hypothalamus (PVN), central nucleus of amygdala (CeA) [35], and nucleus of the solitary tract (NTS) [36] (see Figure 2). Likewise, a different study showed that intestinal anaphylaxis induced important c*-fos *expression in the PVN, NTS, and lateral parabrachial nucleus (LPB) in mice [37]. PVN and CeA are brain areas related to emotional and affective behavior, and they are amongst the main areas containing corticotropin-releasing hormone- (CRH-) expressing neurons. CRH is a key peptide in co-ordinating the behavioral, neuroendocrine, and autonomic responses to stress [38]; being involved in processes of depression and anxiety [39, 40]. Indeed, clinical and animal studies with different CRH antagonists have evidenced antidepressant effects [41–43] and reduction of stress-elicited secretion of cortisol [41]. The activation of CeA [44] and PVN [45, 46] has also been observed in animal models of conditioned taste aversion (CTA), in which animals avoid the consumption of saccharin (conditioned stimulus) after it had been paired with an intraperitoneal injection of lithium chloride (unconditioned, noxious stimulus).

In summary, data on food allergy and nervous system demonstrate that, when forced to ingest the allergen (gavage), allergic animals present activation of emotion-related brain areas and increased levels of anxiety [35]. When the option of drinking or not the allergen solution is offered in a two-bottle preference test, allergic animals avoid drinking the allergen solution and prefer drinking water [32, 35].

## 3. Role of IgE and Mast Cells in Neural Activation

The question that remained to be elucidated is how does the immunological information reach the brain culminating with the neural activation and behavioral change observed? Few studies have focused on this aspect of neuroimmunomodulation; however, some significant findings have been described. The role of IgE in brain activity was determined by the administration of nonanaphylactic anti-IgE antibodies to OVA-sensitized animals. Depletion of IgE prevented c-*fos *activation in the CNS and food aversion in allergic mice [35]. These results highlight the importance of the early phase of immediate allergic response in the neural/behavioral responses observed. IgE-dependent mast cell activation leads to the secretion of preformed mediators (vasoactive amines, neutral proteases), *de novo* synthesized proinflammatory lipid mediators, and the synthesis and secretion of other mediators (growth factors, cytokines, and chemokines) [48]. It has been shown that the pre-treatment of OVA-sensitized mice with a mixture containing antagonists of serotonin, via 5-HT2 receptor (methysergide), and histamine, via H1 receptor (mepyramine), inhibited intestinal edema but food aversion was maintained [32]. The pre-treatment with a glucocorticoid (dexamethasone) inhibited both intestinal edema and food aversion [32]. Similar results were observed in rats [49]. This data suggests that the pharmacological effects of histamine or serotonin are not essential to the development of immunological food aversion. The role of dexamethasone may not be directly correlated to immunological aversion since it is known that corticosteroids may have other immunological, anti-inflammatory [50], and psychological effects [51]. The role of other mast cell mediators in the development of food aversive behavior remains to be determined.

Consistent data demonstrates that mast cells are closely apposed to nerve endings [52–57], giving anatomical support for the role of mast cells in the interaction between immune system and CNS. Neural pathways most likely to mediate this interaction are the autonomic nervous system, via the vagal nerve and sympathetic nerve fibers to the main sites of the immune system, and afferent nerves that convey visceral sensory information to the CNS [58, 59]. Indeed, mediators such as cytokines released by immune cells have been shown to sensitize afferent neurons [60]. In this vein, neonatal treatment with capsaicin, a neurotoxin derived from chilli pepper (plants from the genus *Capsicum*) that promotes a selective dysfunction of sensory fibers such as C-fibers [61], completely blocked c-*fos *expression in the PVN [36] and diminished food aversion in OVA-sensitized mice [62]. The treatment with antagonist of 5-HT3 receptors, expressed in sensory C-fibers [63], diminished the expression of food aversion behavior in sensitized rats [49]. Altogether, these results corroborate the hypothesis that mediators released by mast cell degranulation could stimulate the nerve endings of the C-fibers that, in turn, would transmit the sensory information to the CNS. The presence of IgE receptors (Fc*ε*RI) on sensory neurons in mice has been described [64, 65], and this could represent an alternative way of neuron activation, independent on mast cell or basophils. The implications of the direct SNC activation via IgE-antigen interactions should also be considered in the investigations of the role of neural pathways in allergy.

In the theory of taste aversion, the aversive behavior is related to abdominal discomfort [66]. Animals innately seek pleasure and avoid unpleasant sensations. When motivational conflicts between fundamental goals occur, an animal must either endure unpleasant stimulus to attain pleasure or relinquish pleasure to avoid unpleasant situations. We have approached this question by evaluating the behavior of OVA-sensitized mice when facing a conflicting situation in which the aversive stimulus (allergen) was offered associated with an attractive sweet taste (increasing concentrations of sucrose). We found that food aversion was positively correlated with the levels of OVA-specific IgE and inversely correlated with the animal preference for sucrose sweetened solutions. The aversion behavior was abolished by increasing the sucrose concentration (palatability) of the allergen solution [67]. In a broader scenario, this animal model evidenced a complex crosstalk, in which the very sensorial response triggered by a taste preference could be modulated by an immune response. Thus, food aversion is a behavioral adaptive response resultant of a complex and finely controlled process.

In order to determine the effect of allergic asthma on brain activities, parallel studies investigated allergic aversion behavior in an experimental model of allergic lung disease. Using a dark-light box, it was shown that OVA-sensitized mice, differently from control animals, hesitated entering the attracting and supposedly safer, dark chamber in which the allergen had been previously nebulized, preferring the lit (usually aversive) side of the box. Increased activity of the PVN and CeA was also observed in OVA-sensitized mice following a nasal OVA challenge [47]. 

Using the same experimental model of atopic asthma, it was further demonstrated that the brain and behavior changes observed in OVA-sensitized mice nasally challenged with OVA were (i) IgE dependent, being abrogated by anti-IgE treatment; (ii) mediated by mast cell degranulation, being blocked by the use of sodium cromoglicate (cromolyn, an inhibitor of mast cell degranulation); (iii) not related to airway inflammation, since sensitized C3H/HeJ mice, which did not present pulmonary inflammatory infiltrate, exhibited brain and behavioral changes similar to BALB/c animals [68]. 

Altogether, the findings described above reinforce the fundamental role of the early phase of allergic response on the brain activation and behavior changes associated with avoidance behavior towards allergen exposure. Also, they highlight the sensory function of the vagus nerve in allergic inflammation.

## 4. The Serotonergic Pathway in Airway Allergic Inflammation

Atopic asthma is a chronic inflammatory lung disease mediated by Th2 cells, characterized by airway eosinophilia, airway hyperreactivity (AHR), mucus hyper secretion, and elevated levels of IgE. In addition to the roles of classic mediators of allergic inflammation in asthma-like responses, increasing attention is being given to serotonergic receptors in the airways. Plasma levels of serotonin (5-HT) are elevated in symptomatic asthmatic patients [69]. Moreover, 5-HT receptors (5-HTRs) appear to mediate the secretion of cytokines, prostaglandins, and chemokines by alveolar epithelial cells that may aggravate an already complex inflammatory scenario. The mRNA for several 5-HTRs, such as 5-HTR1, 2A, 4, 6, and 7 (seven-transmembrane domain receptors), and 5-HTR3 (ligand-gated ion channel) have been shown in human type-2 alveolar epithelial cells. 5-HT leads to a calcium-mediated, dose-dependent increase in the secretion of IL-6 and IL-8 [70]. Recently, the expression of several 5-HT2 receptor subtypes has been confirmed in mouse alveolar epithelial cells and macrophages by quantitative PCR [71]. In addition, serotonin binding to 5-HT2C receptors in alveolar macrophages leads to increased expression of CCL2 [71]. In a murine model of OVA-induced asthma-like responses, bronchoconstriction can be mediated by 5-HT2 receptor activation in parasympathetic cholinergic neurons, leading in turn to acetylcholine (ACh) release from nerve terminals and smooth muscle contraction [72]. This points to yet another short-loop neuroimmune interaction mediated by 5-HT in allergic asthma. These data altogether strongly suggest a role of 5-HT in the asthmatic inflammatory responses. The increase of ACh and its consequences in airway inflammation will be further discussed in the following sections.

## 5. The Autonomic Nervous System (ANS) and Immune Responses

The brain and the immune system are hardwired through the autonomic nervous system (ANS), which is composed by the sympathetic nervous system (SNS) and the parasympathetic nervous system (PNS). Description of the innervation of lymphoid organs by the ANS built a solid ground for understanding their implications in health and disease [73]. 

The role of SNS in modulating inflammatory processes is well described [74, 75]. The SNS has pro- or anti-inflammatory functions depending on factors such as neurotransmitter concentration, receptor affinity, timing of SNS activity in relation to the inflammation course, and others [76]. The SNS richly innervates all lymphoid tissues, including bone marrow, thymus, spleen, mucosal-associated lymphoid tissues, and lymph nodes (for review see [77]). The neurotransmitter released by sympathetic nervous pathways is norepinephrine (NE) although adrenergic neurotransmitters released by the adrenal medulla such as NE and adrenaline also modulate inflammation [75].

The expression of adrenergic receptors in cells of the immune system has been thoroughly reported over the past decades [78]. Noradrenaline, adrenaline, and other ligands estimulate alfa and beta cell surface adrenergic receptors with varied affinities and on several cell types. Beta-2 adrenoceptores are the most commonly found amongst adrenergic receptors in almost all cells of the immune system [79, 80], a noteworthy exception being Th2 clones [81]. Decreased density and signaling via these receptors is usually seen at the peak of T-cell activation, which may be relevant to unleashing these cells to their full potential [82, 83]. Additionally, agonist binding to beta-2 adrenoceptors expressed by B cells, natural killer (NK), and macrophages lead to changes in their activity [84–86]. Early evidence of the participation of sympathetic innervation in immunity comes from reports showing a reduction in catecolamine concentration in lymphoid organs following immunization [87]. Several other groups then tackled the issue of NE availability, concentration, and effects during immune responses [74, 88–90]. Innervation by the SNS has been fully demonstrated in all lymphoid organs [91–94].

Evidence for parasympathetic (cholinergic) innervation of the same sites as those described for SNS is more elusive. Cholinergic innervation is undoubtedly present in the thymus and spleen; however, there is no evidence of parasympathetic innervation of the bone marrow and lymph nodes. Nonetheless, it is now clear that non-noradrenergic neurons enter the parenchyma of lymphoid organs, suggesting several other sources of nervous modulation on immunity [95, 96]. The neurotransmitter released by parasympathetic nervous pathways is ACh.

Contrarily to the well-established role of the SNS in disease, the parasympathetic control of inflammation has only been recently described. The anti-inflammatory role of vagal ACh was shown in animal models of pancreatitis [97], inflammatory bowel disease [98], postoperative ileus [99], lethal endotoxemia [100], and hemorrhagic shock [101]. This phenomenon was named “cholinergic anti-inflammatory pathway” [100]. 

Besides the ACh released from parasympathetic nerves, it is relevant to mention that there is increasing evidence for extraneuronal ACh signaling, which has been referred to as a “nonneuronal cholinergic system” [102]. In the airways, non-neuronal ACh producer cells include mast cells, monocytes, macrophages, neutrophils, smooth muscle cells, epithelial cells, and lymphocytes [102, 103]. 

Receptors for various neurotransmitters beyond the sympathetic mediators [79, 104, 105] or parasympathetic [100, 106] are present on immune cells. These neurotransmitters include vasoactive intestinal peptide (VIP), pituitary adenylate cyclase-activating polypeptide [107–109], calcitonin gene-related peptide (CGRP), substance P [110, 111], histamine, and serotonin [112, 113]. Likewise, receptors for neuroendocrine mediators, including CRH [114, 115], *α*-melanocyte-stimulating hormone (*α*-MSH) [115–118], and leptin [115, 119–123] are found on lymphoid tissue. These circuits may be also involved in inflammatory response modulation.

## 6. The Cholinergic Pathway in Airway Allergic Inflammation

Asthma is also associated with increased activity of the parasympathetic nervous system that might underline AHR, one of the hallmarks of asthma. An increase in pulmonary cholinergic nerve activity is associated with asthma, and asthmatic patients are known to be hypersensitive to cholinergic agonists [124]. In fact, the dominant autonomic control of airway smooth muscle in the lungs is provided by the parasympathetic nervous system, and ACh release represents a major bronchoconstrictory pathway. ACh can bind to nicotinic receptors (nAChRs), ligand-gated ion channels comprising 17 different subunits (*α*1–10, *β*1–4, *γ*, *δ*, *ε*) [103, 125–128], or muscarinic receptors (mAChRs), seven-transmembrane G-protein-coupled receptors that comprise 5 subtypes (M1–M5) [129]. The control of ACh release by the vagus nerve involves autoinhibitory muscarinic M2 receptors expressed on the pos-ganglionic nerve fibers [130].

Interestingly, experimental and clinical data on asthma have evidenced dysfunction of muscarinic M2 autoreceptor, which, in turn, contributed to increased release of ACh from airway parasympathetic nerve endings [131–135]. The dysfunction of muscarinic M2 receptor appears to be mediated by eosinophilic major basic protein (MBP), which allosterically blocks muscarinic M2 receptor [130, 136]. The enhanced release of ACh due to M2 dysfunction results in increased airway smooth muscle contraction and mucus secretion via m3AchR present in airway smooth muscle cells and glandular epithelium [137]. Although the increased cholinergic activity of allergic lung contributes to airway flow obstruction, it might have a beneficial effect via the “cholinergic anti-inflammatory pathway.” In this pathway, ACh binds to *α*7nAChR receptor expressed on immune cells such as macrophages, eosinophils, lymphocytes, and dendritic cells [138]. It has been shown that the activation of this receptor attenuates proinflammatory cytokines release by inhibiting NF*κ*B activation, or via activation of Jak2/STAT3 signaling. The later pathway can negatively regulate NF*κ*B binding to DNA or increase the activity of suppressor of cytokine signaling 3 (SOCS3) that results in inhibition of pro-inflammatory cytokine production (for review see [139]). The involvement of nAChRs other than *α*7 subtype by the cholinergic anti-inflammatory pathway is suggested by the study of Matsunaga [140]. These authors proposed a role for *α*4 *β*2 subunits in the downregulation of IL-6, IL-12, and TNF from murine alveolar macrophages after infection with *L. pneumophila *[140]. Moreover, *α*5 nicotinic acetylcholine receptor knockout mice have a more severe experimental colitis than wild-type controls [141]. Finally, it was shown that the vagal inhibition of T-cells proliferation and cytokine release was mediated by an nAChR other than *α*7 [142]. 

Notwithstanding the possible participation of other subtypes of nAChRs in asthma, an* in vivo* model of asthma demonstrated that the activation of *α*7nAChR reduced the numbers of lymphocytes and eosinophils in the bronchoalveolar lavage (BAL) [143]. Experiments with eosinophils obtained from allergic patients demonstrated that activation of *α*7nAChR reduced the production of leukotriene C4 and matrix metalloprotease-9 (MMP-9), mediators related to the pathogenesis of asthma [144].

## 7. Concluding Remarks

In summary, this paper showed that allergic inflammation conveys information to the CNS that, in turn, sends information back to the inflammatory site by releasing neural mediators such as ACh. In asthma, this contributes in smooth muscle contraction (bronchoconstriction) and increased mucus secretion. Similar phenomena occur in the GI tract with increased peristaltism and mucus production. In both cases, these activities that are usually considered as pathologic processes can be viewed as an attempt of the organism to eliminate the irritant stimuli. Therefore, the scenario that emerges from the interaction between immune and nervous systems underscores the robust homeostatic pathways of the brain to allergic inflammation.

## Figures and Tables

**Figure 3:**
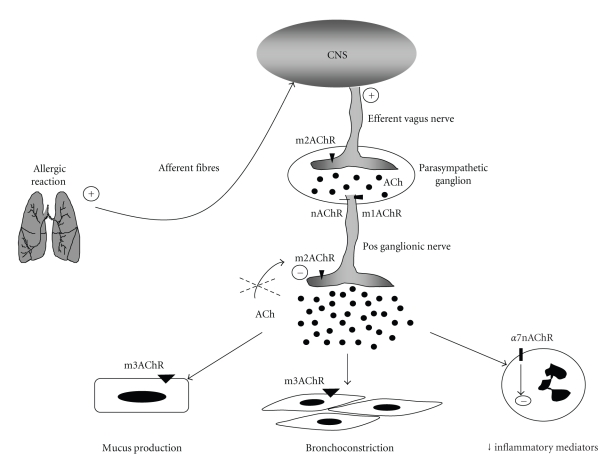
The cholinergic pathways in allergic lung. During allergic reactions, the inflammatory mediators released in the tissue activate the sensory afferent fibers, which convey information to the CNS. The CNS sends information back to the inflammatory site by increasing ACh release from efferent vagus nerve. The neurotransmission in the parasympathetic ganglia is mediated by acetylcholine (ACh) via nicotinic (nAChR) or type 1 muscarinic (m1AChR) receptors. The stimulus generated induces ACh release in the pos ganglionic nerve fiber endings. Type 2 muscarinic receptors (m2AChRs) are autoinhibitory, and the dysfunction of this receptor, observed in allergic asthma, induces increased release of ACh. Increased ACh results in augmented mucus secretion via m3AchR expressed in the glandular epithelium, increased airway smooth muscle contraction (bronchoconstriction) via m3AchR expressed in muscle cells, and decreased inflammatory mediators production via a7nAChR receptor expressed on immune cells.
